# Confusion entre Rosacée et Kératite bactérienne

**DOI:** 10.11604/pamj.2014.18.81.4390

**Published:** 2014-05-24

**Authors:** Rajae Derrar, Rajae Daoudi

**Affiliations:** 1 Université Mohammed V souissi, Service d'Ophtalmologie A, Hôpital des spécialités,CHU, Rabat, Maroc

**Keywords:** Rosacée, kératite, conjonctivite allergique, rosacea, keratitis, allergic conjunctivitis

## Image en medicine

Patient âgé de 38 ans ayant comme antécédent des conjonctivites allergiques a répétition avec à l'examen: une acuité visuelle à perception lumineuse positive, blépharospasme, secrétions purulentes, hyperhémie conjonctivale, ulcère de cornée central de 4 mm à fond propre (flèche blanche A) et à bords infiltrés et hypopion (flèche noire B) Un traitement à base de collyres fortifiés a été entamé avec agents mouillants. Le diagnostic a été redressé suite à la constatation d'une meibomite avec télangiectasies du bord libre (flèche blanche B), d'infiltrats catarrhaux (flèche noire A), d'où l'arrêt des collyres fortifiés et l'instauration d'un traitement reposant sur l'hygiène palpébrale, les agents mouillants et la doxycycline suivi d'injections sous conjonctivales de corticoïdes. L’état du patient s'est amélioré avec disparition des télangiectasies et des infiltrats catarrhaux et début de réépithélilisation de son ulcère (C). Un appel vasculaire sur 360 degrés (flèche bleue D) a été noté, on envisage des injections d'anti VEGF. La rosacée oculaire a une évolution chronique et nécessite un traitement prolongé voire permanent. Il est nécessaire de bien analyser les signes fonctionnels et cliniques afin de ne pas la confondre avec un oeil sec, une conjonctivite allergique ou iatrogène suite à l'utilisation de collyres avec conservateurs ou une kératite infectieuse.

**Figure 1:**
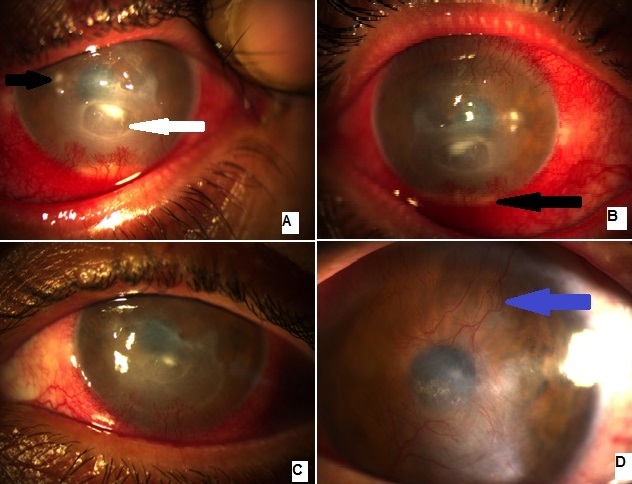
A) ulcère de cornée (flèche blanche) avec microabcès satellites (flèche noire); B)hyperhémie conjonctivale avec hypopion(flèche noire); C) réepithélialisation de l'ulcère; D) cicatrisation de l'ulcère avec appel vasculaire cornéen (flèche bleue)

